# Trimming the genomic fat: minimising and re-functionalising genomes using synthetic biology

**DOI:** 10.1038/s41467-023-37748-7

**Published:** 2023-04-08

**Authors:** Xin Xu, Felix Meier, Benjamin A. Blount, Isak S. Pretorius, Tom Ellis, Ian T. Paulsen, Thomas C. Williams

**Affiliations:** 1 ARC Centre of Excellence in Synthetic Biology and School of Natural Sciences, Macquarie University, Sydney, NSW 2109 Australia; 2 School of Life Sciences, University of Nottingham, Nottingham, NG7 2RD UK; 3 Imperial College Centre for Synthetic Biology, Imperial College London, London, SW7 2AZ UK; 4 Department of Bioengineering, Imperial College London, London, SW7 2AZ UK; 5 Wellcome Trust Sanger Institute, Cambridgeshire, CB10 1SA UK

**Keywords:** Synthetic biology, Biosynthesis

## Abstract

Naturally evolved organisms typically have large genomes that enable their survival and growth under various conditions. However, the complexity of genomes often precludes our complete understanding of them, and limits the success of biotechnological designs. In contrast, minimal genomes have reduced complexity and therefore improved engineerability, increased biosynthetic capacity through the removal of unnecessary genetic elements, and less recalcitrance to complete characterisation. Here, we review the past and current genome minimisation and re-functionalisation efforts, with an emphasis on the latest advances facilitated by synthetic genomics, and provide a critical appraisal of their potential for industrial applications.

## Abstract (web summary)

Naturally evolved genomes tend to be unnecessarily large and redundant, and are not optimised for biotechnological or research applications. In this review, the authors explore genome minimization and re-functionalisation approaches, and discuss their potential for industrial applications.

## Introduction

Modern genomes represent the culmination of ~3.8 billion years of life’s evolution on Earth and encode the stunning complexity we observe in the biosphere. Despite ~70 years of molecular biology research, we are still yet to understand precisely how this complexity is encoded in a genome. Our complete understanding is hindered by two major factors. Firstly, genomes have evolved to facilitate reproduction and survival in diverse environments, through incompletely characterised mechanisms, and thus appear to be incredibly complex to us. Furthermore, under laboratory conditions, a large proportion of genes are individually non-essential^1–4^, yet essential when removed combinatorially. Adding to these challenges is the fact that a significant proportion of genes in any given genome have functions that are yet to be defined. For example, in the genome of *Escherichia coli*, only 48.9% of genes have been characterized, while in the genome of *Saccharomyces cerevisiae*, over 1000 of the ~6000 genes have unknown functions^1,5,6^. To explain this phenomenon, it is hypothesised that genes with unknown functions are either redundant, or their functions are not needed in the lab conditions, and are only important under specific conditions. Secondly, genetic interactions and regulations are overwhelmingly complex, and emergent phenomena are difficult to define in well-studied organisms, let alone rationally designed ones^7,8^. As biotechnological capabilities advance, scientists are not only working to understand the biology, but are also increasingly ambitious in engineering biological systems to solve existing problems. Synthetic biology is a young interdisciplinary field that combines biology with cutting-edge engineering techniques and can benefit agricultural, manufacturing, fuel, environmental and medical sectors. With advances in synthetic biology, complex heterologous pathways have been engineered for wide applications, including the production of value-added molecules, the utilisation of inexpensive nutrition sources and the detection of pollutants or diseases. However, the complexity of biological systems has hindered our ability to modify an existing genome, which might result in genetic incompatibility, instability of the heterologous pathways, and low product yields due to competition for cellular resources. Unexpected results are often obtained after rational engineering, and thus require a laborious trial and error process. As whole-genome synthesis becomes achievable and cheaper, one solution is to unlock a more complete understanding of biology at the genomic level by construction of minimal genomes. Theoretically, a minimal genome consists of the smallest possible number of genes required to support a living cell under a defined set of conditions. Minimal genomes are therefore almost as difficult to define as they are to create, since a minimal gene set will vary according to the environment and construction method. In practice, genome minimisation is more commonly aimed at building a genome with a reduced set of genes relative to its wild-type counterpart, rather than the absolute lowest number of genes. These genomes are intended to be easier to understand and engineer, have fewer uncharacterised genetic elements, and less complex regulatory networks.

**Fig. 1:**
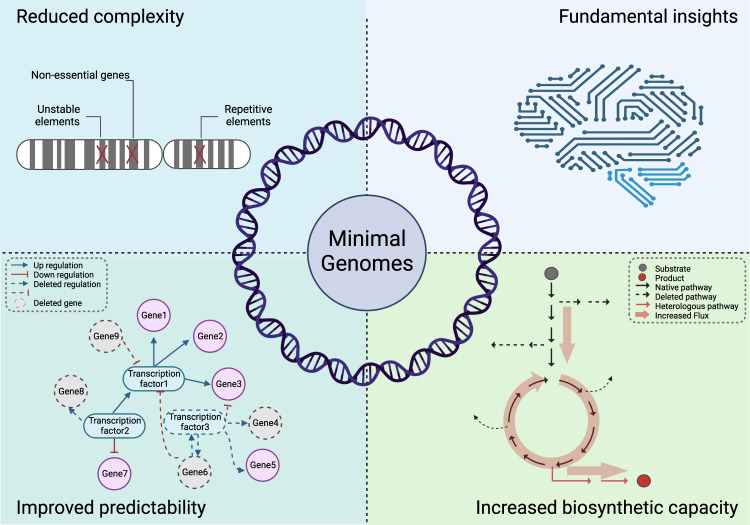
Applications of synthetic and synthetic-minimal genomes. Synthetic genomes provide opportunities to better engineer and understand biology by: reducing the genome complexity by removing non-essential genes and improving genome stability by removing repetitive elements; increasing the predictability of rational design with less complex regulatory and metabolic networks; eliminating unnecessary genes, proteins, and metabolic pathways to free up biosynthetic capacity; and by testing fundamental biological hypotheses by generating new-to-nature genomes for high-throughput analysis.

Engineering minimal genomes will also facilitate a greater understanding of fundamental genome biology. These methods will allow us to understand what constitutes the minimal genome requirement of a functional cell in different contexts. Synthetic minimal genomes will also provide insights into the extent to which genomes can be defragmented and refactored, the roles of non-coding DNA and repetitive elements, and the extent to which global epigenetic regulation can be engineered through genome redesign. Moreover, they can serve as a simplified and superior cell chassis for biotechnological applications due to improved stability, increased predictability via modelling, and greater biosynthetic capacity (Fig. 1).

Here we review past and current genome minimisation efforts, with a focus on the novel genome minimisation strategies enabled by cutting-edge synthetic genomics technologies. Pros and cons of constructing a minimal genome are considered carefully, and the future scope and applications of minimal genomes are discussed.

## Top-down non-synthetic genome minimisation

There are two broad approaches used to generate minimal genomes, termed ‘top-down’ and ‘bottom-up’. ‘Top-down’ minimal genomes are generated by reducing the gene number and genome size of an existing genome.

### Minimisation of an *E. coli* genome

Since the early 2000s, ‘top-down’ genome reduction has been attempted in several bacterial species as well as in the fission yeast *Schizosaccharomyces pombe*^9–13^. These genomes were steamlined via a series of sequential deletions, based on known essential genomic regions and comparative genomics analysis. A selection of previous ‘top down’ genome minimisation approaches and the resulting phenotypes are shown in Table 1. A standard scheme for genome minimization of bacterial systems was also reviewed by Kurasawa et al.^14^ As *E. coli* is the most characterized prokaryotic organism, construction of a simpler *E. coli* cell by genome reduction has drawn great interest. Mizoguchi et al. constructed a 3.62 Mb *E. coli* genome, with a 22.2% genome size reduction compared to the parental strain W3110^11,15^ (Table 1). Specifically, regions of more than ten consecutive non-essential genes were selected as candidates for deletion by comparative genomics analysis between *E. coli* and *Buchnera* sp., which has a small ~600 kb genome and is thought to share a common ancestor with *E. coli*, while essential genes and genes required for *E. coli* growth in the minimal medium were excluded. In addition, transporter genes, insertion sequences (ISs) and toxin–antitoxin pairs were also designed for deletion. In total, 103 candidate regions were selected and deleted individually using lambda-mediated homologous recombination, by which a target region was replaced by a selection cassette, which was subsequently recycled via another round of recombination. The regions that didn’t affect normal growth when absent were then removed in a single strain via 28 cycles of deletions via P1 transduction^15^. This genome-reduced strain, designated as MGF-01, had 1.5 times higher final cell density and a 2.4-fold increase in threonine yield from an engineered pathway compared to the wild-type. With similar approaches, the MGF-01 genome was further reduced by removing the remaining IS sites, generating a 2.98 Mb genome (strain DGF-298)^16^. DGF-298 showed no auxotrophic phenotype and better growth in a medium commonly used in industry, demonstrating its potential for industrial applications.

**Table 1:** Top-down genome minimisation projects

Parental strain and size	Designation and size	Minimisation method	Size/ Percentage of reduction	Phenotypes of the resulting strains	Applications	Refs
*Escherichia coli* W3110 4.65 Mb	*E. coli* MGF-01 3.62 Mb	Lambda homologous recombination and P1 transduction	1030 kb (22.2%)	Improved growth in M9 minimal medium	Enhanced threonine production	^11,15^
*Escherichia coli* W3110 4.65 Mb	*E. coli* DGF-298 2.98 Mb	Lambda homologous recombination and P1 transduction	1670 kb (35.9%)	Better growth fitness and cell yield in a rich medium	N/A	^16^
*Escherichia coli* MG1655 4.64 Mb	*E. coli* CDΔ3456 4.33 Mb	Tn5-targeted Cre/*loxP* excision and P1 transduction	313.1 kb (6.7%)	Normal growth in LB broth	N/A	^9^
*Escherichia coli* MG1655 4.64 Mb	*E. coli* △16 3.26 Mb	Lambda homologous recombination and P1 transduction	1377.2 kb (29.7%)	Much slower growth rate, longer and wider cell shape than the parental strain, abnormal nucleoid organisation	N/A	^17^
*Escherichia coli* MG1655 4.64 Mb	*E. coli* △33a 2.83 Mb	Homologous recombination and FLP-FRT site-specific recombination and P1 transduction	1806.6 kb (38.9%)	Sensitive to oxidative stress	N/A	^18^
*Bacillus subtilis* 168 4.22 Mb	*B. subtilis* Δ6 3.9 Mb	Homologous recombination and excision	320 kb (7.7%)	Normal growth, intracellular carbon metabolism, competence and sporulation, but changed motility	No significant difference of α-amylase (AmyQ) secretion compared to the wild-type	^10^
*Bacillus subtilis* 168 4.22 Mb	*B. subtilis* PG10 2.76 Mb and PS38 2.68 Mb	Homologous recombination and counter selection	PG10 1.46 Mb (36%) PS38 1.54 Mb (36.5%)	Comparable growth to the wild- type in complex medium	PG10 showed substantially higher capacity for protein secretion	^20,54^
*Streptomyces avermitilis* 9.02 Mb	*S. avermitilis* SUKA 7.35–7.51 Mb	Homologous recombination and Cre-mediated recombination	1.52–1.67 Mb (16.9–18.54%)	Could grow on the minimum medium	Higher streptomycin and cephamycin C production than their native hosts; produce the plant terpenoid Intermediate, amorpha-4,11-diene, successfully	^12^
*Schizosaccharomyces pombe* 12.09 Mb	*S. pombe* A8 11.43 Mb	Homologous recombination by the Latour method	657.3 Kb (5.43%)	Decreased uptake of glucose and some amino acids compared with the parental strain	2.7-fold increase in the concentration of cellular adenosine triphosphate; 1.7- and 1.8-fold increase in the production of green fluorescent protein and secreted human growth hormone, respectively	^13^

In an earlier study, Hashimoto et al. reduced the genome of *E. coli* MG1655 from 4.64 Mb to 3.26 Mb^17^ using lambda homologous recombination and P1 transduction (Table 1). However, the minimised strain △16 had a much slower growth rate, and abnormal cell shape and nucleoid organisation. A further 430 kb was then deleted, yielding *E. coli* △33a with the smallest *E. coli* genome reported so far (2.83 Mb)^18^. It was shown that △33a was sensitive to oxidative stress, which might preclude its use in industrial fermetation settings without further modification. From the studies above, we note that the phenotypes of minimised *E. coli* strains are different in each study. This likely results from the different engineering approaches, specific regions of deletions, and the mutations arisen during the construction.

### Minimisation of *Bacillus subtilis*, *Streptomyces avermitilis* and *Schizosaccharomyces pombe* genomes

*Bacillus subtilis* is a model Gram-positive bacterium with gene essentiality now well characterised^3,4,19^. In the first study of *B. subtilis* genome minimisation, prophages and AT-rich islands were removed by homologous recombination, producing a strain with 7.7% genome reduction (strain Δ6)^10^ (Table 1). The genome-reduced strain had normal growth, and comparable heterologous protein production and secretion compared to the wild-type. With more gene functions discovered, the regions not required for cell survival in the rich medium were selected and deleted stepwise, and a 36.5 % decrease in genome size was achieved (strain PS38)^20^ (Table 1). The genome-minimised *B. subtilis* had comparable growth rate to the wild-type in a rich medium. Interestingly, unknown genes still represented 18% of the total genes in PS38, while the proteins of unknown function only represented 2.5% in the total expressed proteome. The finding suggested that the unknown genes were poorly expressed generally and might only be useful under specific conditions. It is therefore possible the *B. subtilis* genome could be further reduced by removing a large proportion of genes with unknown functions.

*Streptomyces avermitilis* is an industrial microorganism able to produce various secondary metabolites. A sub-telomeric region >1.4 Mb, which did not contain essential genes, was deleted sequentially from the 9.02-Mb linear chromosome of *S. avermitilis*. This generated a series of deletion mutants, whose sizes corresponded to around 80% of the wild-type chromosome^12^ (Table 1). After integration with gene clusters encoding the production of streptomycin and cephamycin C, the deletion mutants produced both antibiotics at higher levels than the natural hosts.

In an example of eukaryotic genome reduction, 657.3 kb was deleted from the terminal regions on chromosomes I and II of the fission yeast *Schizosaccharomyces pombe* by the Latour method^21^, which included integration of a *ura4* + marker by homologous recombination and subsequent counter-seletion for deletion of the inserted *ura4* + marker using 5-FOA medium^13^ (Table 1). The resulting strain had decreased uptake of glucose and some amino acids, but had increased levels of heterologous protein production.

These ‘top-down’ genome minimisation or reduction studies have improved our understanding of fundamental biology, and showed that reduction of genome size does not necessarily generate strains with impaired fitness. Some genome-reduced strains have similar or even superior fitness and industrially favourable phenotypes relative to their wild-type parents. These top-down approaches are straightforward, generally affordable, and are often the method of choice for generating a small number of deletions, or deletion of a large non-essential gene cluster. However, for genome-wide minimisation, the reduction process can be very challenging. For example, tens or even hundreds of rounds of transformation might be required to yield and identify a strain with significant genome reduction. In addition, the deletion regions were limited to the segments of genes with characterised functions. Unknown genes, intergenic regions, introns, and non-annotated genomic features were not targeted for deletion. Moreover, the target regions for deletion were chosen based on the essentiality of single genes, while genetic interactions and synthetic lethality were not taken into consideration. Thus, it is very difficult to generate a minimal genome for practical applications using previous non-multiplexed gene knock out approaches. A faster and more systematic approach is needed for genome-level minimisation and eventual minimisation.

## Synthetic genomics unlocks new possibilities for genome minimisation

With the decreased DNA synthesis costs and the development of large-scale DNA assembly techniques, it is now possible for ‘bottom-up’ genome minimisation and re-functionalisation via whole-genome design and synthesis. These ‘bottom-up’ approaches rely on either the de novo synthesis of a new genome, or the stepwise replacement of an existing genome with rationally designed and chemically synthesised DNA.

### Synthesis of *Mycoplasma* genomes

**Fig. 2:**
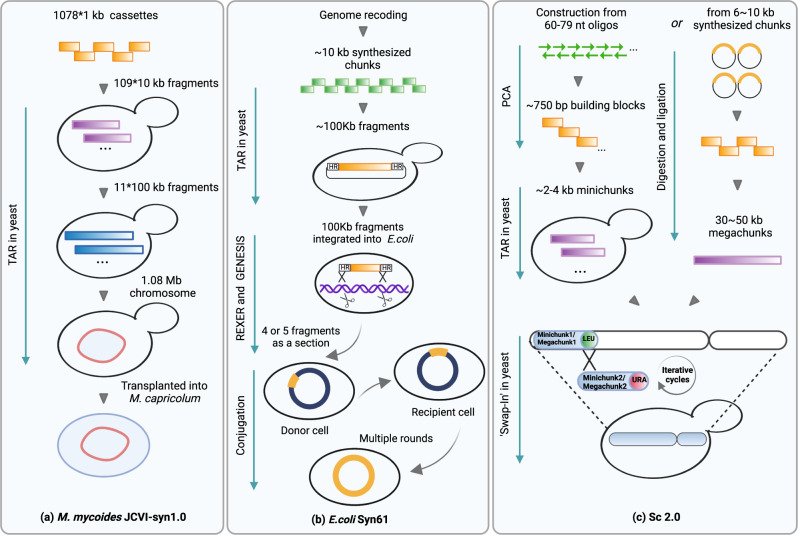
Construction of synthetic genomes. Synthetic oligonucleotides are assembled into progressively larger double-stranded DNA fragments using methods such as polymerase cycling assembly (PCA), digestion and ligation, and transformation-associated recombination (TAR) in yeast. **a** The synthetic *Mycoplasma mycoides* genome was assembled via TAR in yeast from 1 kb DNA cassettes gradually into larger fragments (10 kb and 100 kb), and finally into 1.08 Mb genome, prior to extraction and genome transplantation into *M. capricolum* to generate a new *M. mycoides* strain controlled by the synthetic genome (JCVI-syn1.0). **b** Genome-wide recoding was conducted to generate an *E. coli* strain with 61 codons. The recoded genome was assembled from 10 kb fragments into 100 kb fragments on a bacterial artificial chromosome via TAR in yeast. A 100 kb fragment was integrated into different *E. coli* strains by replicon excision for enhanced genome engineering through programmed recombination (REXER), and 4–5 100 kb fragments in total were assembled as a big section by genome stepwise interchange synthesis (GENESIS). These large DNA sections were assembled into a whole recoded genome through a conjugation-based strategy, which generated Syn61. **c** The Sc2.0 was initially constructed from 750 bp building blocks, and through PCA and TAR in yeast into 2–4 kb minichunks; or in more-recent Sc2.0 chromosomes, assembled from 6–10 kb chunks into 30–50 kb megachunks via in vitro digestion and ligation. The minichunks or megachunks were then transformed into yeast cell to replace the native genome iteratively.

In 2002, the poliovirus cDNA (~7.5 kb) was the first to be chemically synthesised^22^. Following the success of the synthetic poliovirus genome, Gibson et al. synthesized the first prokaryotic genome, a 582,970–base pair genome of *Mycoplasma genitalium*, which is a bacterium with the smallest genome grown in pure culture^23^. Since *M. genitalium* has a very slow growth rate, two faster-growing *Mycoplasma* species were selected for subsequent research. *M. mycoides* was chosen for de novo genome synthesis, and *M. capricolum* as the recipient cell for the synthetic *M. mycoides* genome. The synthetic genome of *M. mycoides* (JCVI-syn1.0) was successfully completed by Gibson et al. in 2010 with four watermark sequences, designed to differentiate the synthetic genome from the wild-type genome^24^. The synthetic genome was assembled by transformation and homologous recombination in yeast of 1078 overlapping 1 kb DNA cassettes into a 1.08 Mb genome, and then transplanted into a *M. capricolum* recipient cell to create a new synthetic strain showing a similar phenotype to *M. mycoides* (Fig. 2a).

### Synthesis of a minimal *Mycoplasma mycoides* genome

**Fig. 3:**
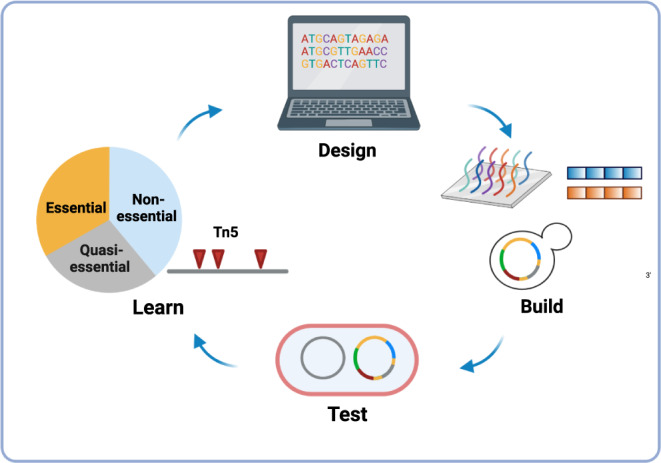
‘Bottom up’ construction of a minimal *Mycoplasma mycoides* genome (JCVI-syn3.0). The minimal genome was first designed based on existing genomic knowledge; the synthesis started from oligonucleotides and gradually assembled into a genome via methods such as polymerase cycling assembly (PCA) and transformation-associated recombination (TAR) in yeast; the genome was extracted from yeast and transplanted into a *M. capricolum* recipient cell to test its growth. At the end of each cycle, gene essentiality in the resulting strain was re-evaluated by Tn5 mutagenesis. In total, four Design-Build-Test-Learn cycles were conducted to produce a viable strain with its genome smaller than any autonomously replicating cell in nature (JCVI-syn3.0).

In a following study, the team applied whole-genome design and synthesis to minimise the *M. mycoides* genome^25^ (Fig. 3). The minimal genome was initially designed according to existing transposon mutagenesis data and molecular biology knowledge. However, the initial design did not generate a viable strain. Subsequently a global Tn*5* mutagenesis study was conducted to determine the essential genes, non-essential genes, alongside quasi-essential genes. Quasi-essentiality, a concept developed during the design of the minimal synthetic *Mycoplasma* genome, describes genes whose deletion wouldn’t result in cell-death immediately, but would cause minimal to severe growth impairments. Whilst not being strictly essential, these genes are needed for long term fitness^25^. By retaining quasi-essential genes and avoiding deleting synthetic lethal pairs, a viable minimised genome was obtained (JCVI-syn2.0). In addition, 42 more genes were removed after another round of Tn5 mutagenesis in syn2.0, yielding an approximately minimal genome (JCVI-syn3.0) with removal of 428 genes in total. The syn3.0 strain has a genome of 531 kb, smaller than any autonomously replicating cell known in nature, has 51% genome reduction compared to syn1.0 and a doubling time of ~180 min, which is slower than of syn1.0, which has a doubling time of ~60 min. However, its growth is much faster than the 16-h doubling time of *M. genitalium*. It can be inferred that there is a trade-off between removing as many genes as possible and maintaining a certain level of growth fitness. In a follow-up study, adaptive laboratory evolution (ALE) was conducted to improve the growth rate of JCVI-syn3.0 by 15%^26^.

### Synthesis of an essential *Caulobacter crescentus* genome

Chemical synthesis has also been applied to rebuild the genome of *Caulobacter crescentus*, a model system for cell cycle, cellular differentiation, and cell division studies. This synthetic *C. crescentus* genome, *C. eth-2.0*, was designed to only contain essential genes^27^. Initially, a 785,701-bp genome was designed computationally according to characterisation data from transposon gene knockout studies. However, the natural sequences failed to be commercially synthesized due to synthesis constraints such as homopolymers and repetitive sequences. Thus, computational DNA design algorithms were applied and resulted in 10,172 base substitutions to facilitate DNA synthesis. In addition, 123,141 base substitutions were introduced within protein-coding sequences to reduce the number of hypothetical genetic elements from 6290 to 799. These removed elements included alternative open reading frames (ORFs), transcriptional start sites (TSSs) and ribosome binding sites (RBSs) within the coding sequences (CDSs). In total, 56.1% of all codons were replaced by synonymous codons in *C. eth-2.0*.

The *C. eth-2.0* genome was constructed from the assembly of 236 DNA blocks of 3-4 kb into 37 large segments of 19–22 kb, and further into 16 ‘mega-segments’, and finally into the full-length chromosome via transformation-associated recombination (TAR) in yeast. Functional analysis showed that 81.5% of all synonymously recoded essential genes had no significant influence on their functionality, which demonstrated the potential of synonymous recoding to facilitate de novo genome synthesis. This analysis also revealed that 98 genes lost their function due to rewriting, which may have been a result of inaccurate annotations causing other important features to be modified.

The fully assembled *C. eth-2.0* failed to replace the native genome and generate a living cell. A follow-up study compared the transcriptional profiles of genes expressed from plasmid-borne *C. eth-2.0* segments to those on the native *C. crescentus* genome, with the intention of uncovering important elements that had been disrupted by synonymous recoding. The analysis resulted in 60 promoter annotations being refined and showed that in *C. eth-2.0*, 18 termination elements and 77 transcription start sites had been unintentionally introduced^28^. Translational regulations for 20 CDSs and an essential translational regulatory element for the expression of ribosomal protein were also identified^28^.

### Synthesis of a recoded *E. coli* genome

Advances in synthetic genomics have also facilitated global codon reassignment. Total synthesis was implemented in *E. coli* for genome-wide removal of three codons, generating a synthetic *E. coli* genome with 61 codons^29^. In the synthetic *E. coli* genome, two of the serine codons and a stop codon were replaced, and an essential transfer RNA gene was freed up. 10 kb synthetic constructs were assembled into 37 fragments of around 100 kb each onto bacterial artificial chromosomes (BACs) by TAR in yeast (Fig. 2b). The 100 kb synthetic fragments were integrated into different *E. coli* strains in parallel, by ‘replicon excision for enhanced genome engineering through programmed recombination’ (REXER), which used CRISPR-Cas9 and lambda-mediated recombination to replace the genomic DNA with the recoded DNA from BACs. Seven strains with partially synthetic genomes were generated by integrating four or five fragments of around 100 kb via consecutive REXER cycles with alternating uses of positive- and negative-selection, enabling genome stepwise interchange synthesis (GENESIS) (Fig. 2b). The recoded large sections were at last combined into a full synthetic recoded genome by conjugative transfer and recombination, designated as ‘Syn61’. The resulting codon compressed strain Syn61 provides huge potential for production of proteins with novel functions via codon reassignment, as well as for industrial bioprocesses since they are resistant to phage contamination through genetic code incompatibility. This was demonstrated in a later study where the three previously freed-up codons were reassigned to enable the incorporation of non-canonical amino acids. Cells with reassigned codons were resistant to viral infection and were able to produce novel polymers and macrocycles^30^. However, it is very challenging to free up more codons since large scale genome recoding will not only increase the technical difficulty of DNA synthesis and assembly, but also affect GC content, protein expression, and global epigenetic signals, potentially resulting in severe fitness-defects or lethality^31^. Ostrov et al. reported the design, synthesis, and testing progress of a 57-codon *E. coli* genome in 2016, in which they have validated the function of 63% of recoded genes, and they are still working on the assembly of the fully recoded strain^31,32^.

### Synthesis of a *S. cerevisiae* genome

In parallel to the success of de novo synthesis of bacterial genomes, a global consortium led by Jef Boeke at New York University has been pursuing an ambitious project Sc2.0, aiming to build the first synthetic eukaryotic genome, that of *S. cerevisiae*. The aim of the project is not only to gain insights into yeast genomics, but also to create a simpler version of a yeast cell with comparable fitness to wild-type, which could be streamlined and refactored for different engineering purposes. The following changes were implemented in the design of Sc2.0: unstable or redundant elements including retrotransposons, subtelomeric repeats and introns were removed; the repetitive transfer RNA (tRNA) genes were relocated to a ‘neo-chromosome’ to test their functions and stability separately; TAG stop codons were swapped with TAA for future codon reassignment; native telomeres were replaced with a standardised synthetic version; strings of codons were recoded to synonymous codons as ‘PCRtags’, which can be used as watermarks to distinguish the synthetic sequences and wild-type sequences^33,34^. Initially, the construction of synthetic chromosomes started from oligonucleotide assembly into 750 bp building blocks, then assembled in yeast to produce minichunks^34^ (Fig. 2c). Several overlapping DNA minichunks, with an auxotrophic marker (*LEU2* or *URA3*) in the last minichunk, were co-transformed in yeast cell to replace the native DNA^35^. The integration of the next group of minichunks would over-write the previous marker, enabling both positive and negative selection by the ‘SWAP-In’ method^35,36^ (Fig. 2c). Ultimately, this would generate a complete synthetic chromosome. Most recently, chromosome assembly has been further expedited by starting with 6–10 kb commercially synthesized ‘chunks’. Four or five chunks were ligated in vitro for the assembly of a 30–50 kb megachunk, before integration into yeast with an auxotrophic marker. The megachunks were ‘SWAPPED-In’ gradually to create a synthetic chromosome (Fig. 2c). Thus far, the construction of all synthetic chromosomes is close to completion, with nine strains containing one synthetic chromosome reported to have comparable growth with the wild-type strain^37–45^. The global team is on track to build an entirely synthetic yeast genome. Once completed, the synthetic genome will have a nearly 8% genome size reduction, and will serve as a whole-genome diversification and minimisation platform^36^.

‘Bottom-up’ genome construction enables implementation of novel design changes at the whole-genome level. However, it is still currently very costly for genome-scale synthesis, especially for eukaryotic genomes. There will also be regions difficult to synthesise that require recoding. As more knowledge of genome biology and gene regulation is gained through the study and rewriting of genomes, our inability to design a functional minimal genome from scratch is continually highlighted. However, the ensuing iterative design, build, test and learn cycles needed to generate a final functional minimal genome will ultimately refine our understanding and capabilities in genome design^25,27,45,46^.

## Sc2.0 SCRaMbLE: ‘bottom-up’ genome engineering meets top-down pruning for genome minimisation

**Fig. 4:**
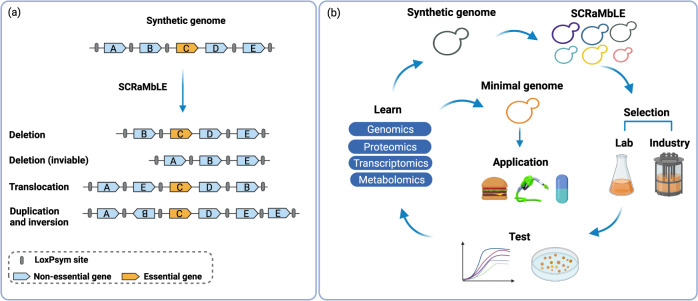
Genome minimisation by SCRaMbLE. **a** Genome diversity generated by SCRaMbLE. LoxPsym sites are inserted in the 3’UTR of all non-essential genes. Upon induction of SCRaMbLE, various genome rearrangement events can happen, for example, deletion of non-essential gene ‘A’, translocation of ‘E’ and ‘B’, inversion of ‘B’ and duplication of ‘E’. However, deletion of a gene-cassette containing essential gene ‘C’ leads to cell death. **b** Creation of minimal and industrial-minimal genomes via SCRaMbLE. Genome rearrangements including deletions of large segments can occur upon activation of SCRaMbLE. Applied with appropriate selection pressures, the SCRaMbLEd cells with reduced genome size can be selected under lab conditions, and under industrial conditions in parallel, such as different carbon source utilisation, stress conditions, and etc. The selected strains are then tested for phenotypes and analysed by ‘omics’ approaches, which would shed light on future rational design of minimal genomes. Iterative rounds of SCRaMbLE-Selection-Test-Learn can be applied before the generation of minimal genomes or industrial minimal genomes, which can serve as a simplified chassis for industrial engineering.

In the wild-type *S. cerevisiae* genome, most of the genes are individually non-essential, and many genes have homologues originating from historical duplication events. This indicates a great potential for genome minimisation. Complementing these existing factors, a novel genome diversification and minimisation approach has been designed into the Sc2.0 genome. In the synthetic genome, all non-essential genes and major landmarks have been designed to be flanked by the symmetrical loxPsym recombination sites. This enables the most dramatic novel ability of Sc2.0 strains, the Synthetic Chromosome Rearrangement and Modification by LoxPsym-mediated Evolution (SCRaMbLE) system. Upon induction of the SCRaMbLE system by an active Cre-recombinase, an effectively infinite number of genome rearrangements can be generated, including gene deletions, duplications, inversions, and translocations between any two loxPsym sites (Fig. 4a). The ability of SCRaMbLE to generate genome diversity had been confirmed in a previous study, in which 156 deletions, 89 inversions, 94 duplications, and 55 additional complex rearrangements were identified from deep sequencing of 64 synIXR SCRaMbLEd strains^47^. Moreover, each SCRaMbLEd strain has a unique genome. Since deletions are the most frequent recombination events arising via SCRaMbLE, it can generate a near-infinite number of variable reduced genomes that are sufficient to keep the cells alive and functional. Compared to previous genome reduction approaches, SCRaMbLE-assisted minimisation can be achieved by sequential ‘bottom up’ genome synthesis followed by ‘top down’ SCRaMbLE-mediated genome reduction approaches.

One major challenge involved in applying the Sc2.0 SCRaMbLE system for genome minimisation is the loss in cell viability after SCRaMbLE, which decreases the chances of long sequence deletions. This is because although loxPsym sites were inserted 3 bp downstream of the stop codon of non-essential genes, in many cases, there are essential genes and non-essential genes present between two sequential loxPsym sites. As a result, these non-essential genes have to be deleted by SCRaMbLE together with the adjacent essential genes, leading to loss of viability. Fortunately, a complementary ‘bottom-up’ approach can address this challenge by introducing a minimal-essential chromosome containing all individually essential genes but without loxPsym sites so that the extra copy of the essential genes can be stably maintained during SCRaMbLE. This principle has been tested via SCRaMbLE-ing of synIII and the synXII left arm (synXIIL), which demonstrated the capacity for deletion of large regions containing the essential genes, now complemented by the supplemental copies^48,49^. With an extra copy of all essential genes from the genome, this would increase the post-SCRaMbLE population viability, and increase the probability of finding strains with smaller genomes. Even in this scenario, there is still likely to be a loss in population viability through synthetic lethality, where the loss of individually non-essential genes in combination causes lethality^49^.

After SCRaMbLE, there will be a mixed population of genomes, except for the reduced genomes, some without changes, some with a net increase in genome sizes, and some with undesired or deleterious rearrangements. Thus, the other challenge for SCRaMbLE-assisted minimisation is how to identify SCRaMbLEd strains with reduced genomes efficiently. One approach is to select deletions by integration of marker genes. *URA3* insertion and 5-FOA counterselection was successfully applied to compact the synthetic chromosome XII left arm^48^. With the aid of an essential gene array, 64 kb of a total 170 kb was deleted in syn XIIL via only one round of SCRaMbLE-based genome compaction (SGC)^48^. After another two rounds of SGC, a strain with 58% reduction of synXIIL (~100 kb deleted from 170 kb) was generated and had comparable growth with wild-type strain. This study has demonstrated SCRaMbLE is an efficient system for yeast genome minimisation. However, selection of deletion at a specific locus does not rule out the possibility of duplications at another locus. Another approach we propose is to determine genome sizes assisted by fluorescence-activated cell sorting (FACS). Non-lethal double-strand DNA-specific dyes are able to sort out the cells with different genome sizes (preliminary data from our group), and can be used to stain and sort out the SCRaMbLEd cells with smaller genomes. After staining, FACS could then be applied for high-throughput screening of reduced genomes. This approach would enable the screening of 2000–5000 cells per second for their approximate genome size, easily providing the throughput necessary to find cells with rare large deletions. The phenotypes of sorted strains could then be tested, and analysed using systems biology approaches, which will shed light on the genetic elements that are common to minimal yeast genomes, and suggest paths towards rational genome design. Iterative rounds of SCRaMbLE, selection, test and learn could be conducted to explore the compactability of the yeast genome (Fig. 4b). Without the need to identify the essentiality of each genetic element, multiple rounds of SCRaMbLE-based selection provide an evolutionary process to enrich cells with smaller, and eventually minimal genomes.

In previous studies, minimal genomes were constructed in one specific condition, usually in rich media, which might not be useful for industrial settings. This, in-part, has led to pervasive arguments that minimised genomes cannot be industrially relevant. However, the SCRaMbLE-based minimisation process provides the opportunity for ‘industrial minimal’ genome selection by carrying out the iterative minimisation process with outgrowth under industrially relevant conditions, or with alternative selection pressures to co-select for desired industrial traits along with minimisation. This approach would reflect the fact that while there may be only one truly minimal genome, there are likely to be near infinite possibilities for genomes that are simultaneously minimised and selected for other desirable traits. Such industrial minimal genomes could be co-selected for phenotypes such as temperature tolerance, stress tolerance, or the bioproduction of specific proteins and metabolites.

## Genome minimisation: does the ends justify the means?

Despite the applications of cutting-edge synthetic genomics and engineering approaches, genome minimisation projects are still relatively costly and time consuming. Thus, there is ongoing debate as to whether it is cost effective to build a minimal genome.

One school of thought posits that construction of minimal genomes can bring significant impacts for research and industrial purposes. First of all, it facilitates a deeper understanding of functions and interactions of genome components, and uncovers knowledge of how a genome is programmed into a living and functioning cell. In the redesign and chemical synthesis of *C. eth-2.0*, 52 instances of inaccurate annotations of the *Caulobacter* genome were identified via analysis of non-functional genes, and 27 regulatory elements within protein-coding sequences were discovered^27^. To construct a minimal genome of *M. mycoides*, gene essentiality was re-identified by whole-genome Tn5 mutagenesis, and a class of quasi-essential genes, which do not result in lethality directly but are required for robust growth, were identified and retained in JCVI-syn3.0^25^. Reorganisation of essential genes from Sc2.0 chrIII had little effect on their transcriptional level despite altered gene order and orientation, demonstrating the feasibility of defragmentation and reorganisation of the yeast genome^49^.

Secondly, reducing the genome size can improve fitness or biomass yield possibly by avoiding unnecessary energetic costs. *E. coli* MGF-01 with 1030 kb removed had improved growth in M9 minimal medium^11,15^. The reduced genome *E. coli* strain DGF-298, with 1670- kb deleted, had better growth fitness and cell yield in a rich medium than the wild-type strain^16^. In addition, mobile elements, recombinogenic and repetitive DNA are often deleted, which leads to better stability and more efficient and predictable genetic modifications^37,50^. The *E. coli* multiple-deletion (MDS) series with removal of IS elements was subsequently free of IS-mediated mutagenesis, thus enabling more stable propagation of recombinant genes and plasmids^50^. The MDS42 strain also showed more than 180-fold higher efficiency of DNA transformation by electroporation of a 2.7 kb pUC plasmid than its parental strain, which is comparable to, or even better than, the efficiencies of commercial competent cells^50^. Further deletions were made from MDS42 to generate commercial strains ‘Clean Genome® *E. coli*’ by Scarab Genomics, which serves as a superior host for protein and nucleic acid production^51,52^. With major IS elements deleted in *Corynebacterium glutamicum*, improved production of recombinant proteins was observed possibly due to the increased stability of plasmids^53^. Thirdly and more importantly, minimal genomes could serve as better chassis cells for industrial applications. Genome minimisation is likely to reduce physiological complexity and therefore make metabolic modelling more predictive and systems biology more informative. Engineered heterologous pathways will also be less likely to be affected by complex native metabolism. In theory, minimal genomes will only contain the smallest set of genes required for survival and replication within a given environment, and their biosynthetic capacity will therefore be liberated to produce desired proteins and metabolites. This concept is supported by several previous studies. For example, an increase in threonine production was shown in the genome-reduced *E. coli* strain MGF-01^11^, *S. avermitilis* with more than 1.4 Mb deletion enabled higher streptomycin and cephamycin C production than their native hosts, and *B. subtilis* PG10, with a 36% genome reduction, showed substantially higher secretory protein production^54^. Furthermore, SCRaMbLE of synthetic chromosomes in yeast has been utilised to increase the yields of several valuable compounds including carotenoids, aromatics and antibiotics^55–57^. Although these SCRaMbLEd strains did not have reduced genomes, SCRaMbLE has been used previously to streamline individual chromosomes^48,49^, making the use of SCRaMbLE to simultaneously streamline genomes and improve biosynthetic capacity an intriguing near-term possibility.

In contrast to the optimistic prospect of higher biosynthetic capacity and reduced complexity in genome-reduced strains, another school of thought maintains that minimal genomes are of little value to industrial applications. It usually takes a long-time to assemble or engineer a minimal genome, followed by a lengthy trial and error process to return a minimal genome strain to comparable fitness and function with the wild-type strain. It is also worth noting that some of the minimised genomes reported thus far are probably not industrially applicable, nor were they designed to be. For example, the *M. mycoides* JCVI-syn3.0 grew slowly, and its metabolism is extremely reduced^25,58^. The genome-reduced *E. coli* △16 had a severe growth defect and abnormal nucleoid organisation, while △33a with a genome of only 2.83 Mb was sensitive to oxidative stress^17,18^ In addition, a minimal genome is affected by the conditions under which it is constructed. Currently, most minimal genome projects are constructed in nutrient-rich medium. The genetic elements encoding stress response and tolerance are often discarded, which might result in decreased fitness under industrial fermentation conditions. Furthermore, the resulting minimal genome strain might not have intermediates or co-factors for expressing a heterologous pathway.

The shortcomings mentioned above could be overcome via ALE or genetic engineering to either select for the mutations to overcome the stress, re-insert the required genes, or select for their function during a ‘top-down’ minimisation process. ALE is an effective approach that has been demonstrated to improve the fitness of JCVI-syn3.0, a recoded *E. coli* genome, as well as the synXIV strain from Sc2.0^14,26,45,59^. Another approach is to build customised neo-chromosomes or entire minimal genomes for different requirements, such as for different fermentation conditions or for producing different categories of compounds. Sc2.0 is constructed based on the genome background of laboratory strain S288c, which lacks genetic diversity compared to industrial yeast strains. To address this, Kutyna et al. constructed a synthetic pan-genome neo-chromosome (PGNC), which incorporated 75 predicted ORFs from industry, human pathogen and natural isolates^60^. With the presence of PGNC, the resulting strain was able to utilise a wider range of carbon sources beyond the Sc2.0 parental strain. This is a clear example of how construction of neo-chromosome can improve the industrially favourable features and expand the applications of the synthetic strain. However, given the current state of biological knowledge, much more effort is required to realise this idealised view of synthetic genome design. Given the large amount of work to construct industrial applicable minimal genomes, optimisation of the pathway via genetic engineering directly is clearly a more straightforward and promising approach in the immediate future. However, the weight of evidence from the existing literature suggests that minimised genomes hold great promise for both understanding biology and engineering superior industrial strains.

## Conclusion and future prospects

Overall, genome minimisation and re-functionalisation are very attractive research topics. The key current bottlenecks for the construction of novel minimal genomes are the workload and cost required to assemble comparatively small synthesised DNA chunks into ever-larger fragments, transplant these into host organisms, and modify and ‘debug’ these synthetic genomes^46,61^. Enabling technologies such as enzymatic DNA synthesis, together with automated DNA assembly platforms can greatly reduce the cost of synthesis, as well as shorten the time and labour required for genome synthesis projects. Therefore, it could become economically viable to create minimal genomes for a wide range of species that are specific to particular applications and enable rapid iteration in design-build-test cycles^62,63^.

However, the increasing dominance of the ‘bottom-up’ approach via synthesis shouldn’t be taken for granted. The ever-expanding ecosystem of CRISPR-based tools for genome editing has recently started to produce new methods for rapid and precise removal of large regions of genomes, especially those of mammalian cells. In particular, the Prime Editing system of Liu and co-workers can insert pairs of site-specific recombinase sequences, allowing a targeted genome section to be cleanly deleted by a recombinase^64,65^. Two variations of Prime Editing, called PRIME-Del and PEDAR go further, doing precise programmed deletions over 10 kb at a time without the need for any recombinase^66,67^. Multiplexing these methods to allow many large deletions from a genome at the same time is the next challenge, but such multiplexing has already been shown to be possible for Prime Editing’s precursor, Base Editing, where over 13,000 edits in a human genome can be achieved in a cell in parallel^68^. For large genomes such those for mammalian cell lines, multiplex CRISPR-guided precise deletions are likely to be the quickest and cheapest route to minimal genomes, and will hopefully ensure that this important set of organisms for industrial biology and research do not miss out on the many opportunities that minimal and synthetic genomes can offer.

**Fig. 5:**
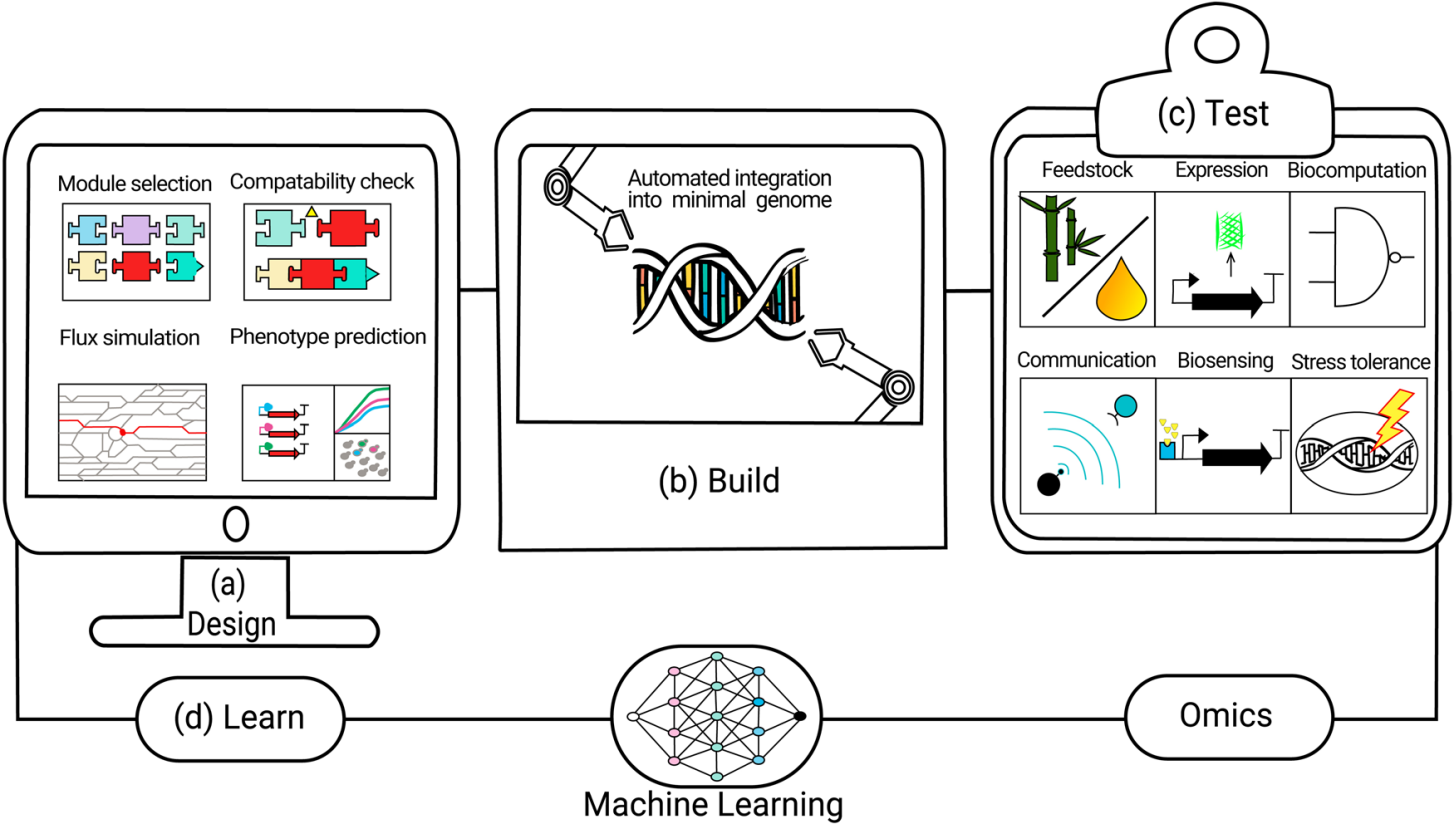
Future smart design, construction, and applications of minimal genomes. Synthetic minimal genomes will facilitate the realisation of some of synthetic biology’s founding principles of modularity, computational design, automated building, and data-driven feedback into rational design iterations. **a** In the design stage, modules are selected from a database containing a variety of functionalities, such as carbon source utilisation, biosynthetic pathways, biosensing and growth-regulating modules. Modelling can then be used to ensure compatibility between modules, simulate metabolic flux, fine tune expression levels, and predict phenotypes or the yields of products. **b** Assisted by robotic high-throughput cloning and assembly techniques, modules are constructed and integrated into chassis minimal genomes. Multiplexing for design variations and integrations into different minimal genome strain contexts could allow for further optimisation. **c** The resulting strains could gain versatile functions, from growing on non-conventional feedstocks to producing recombinant proteins, and biosensing. Ultimately, designed minimal-genome strains could be analysed by multi-omics approaches. **d** The resulting data could feed back to machine learning models, enabling a better understanding of minimal-genome rational design principles, and possibly, using generative AI, design new-to nature minimal genomes.

Rapid and inexpensive generation of streamlined genomes can lead to a diverse set of context-dependent minimal genomes. Systems analysis of both fundamental and applied minimal genomes would provide a rich repository of omics data for design guidelines for future simple and more robust genomes, and even de novo new-to-nature genomes^69^. A minimal genome could function as a platform for modular plug-and-play integration of metabolic pathways, such as ‘feedstock modules’ for efficient utilisation of non-conventional carbon sources, ‘production modules’ for making different groups of compounds, ‘stress and toxic compound resistance modules’, ‘biosensing modules’ for in vivo metabolite measurements and feedback control, ‘cell-to-cell communication modules’, ‘data-storage modules’ and ‘bio-computation modules’ (Fig. 5). Each module could also be optimised via ALE separately and subsequently reintegrated while leveraging reduced genomic complexity for troubleshooting. This could allow for interoperability of modules when relying on the same minimal genome strain and enable a greater degree of orthogonality when designing each individual module. As we uncover the minimal genetic modules required for different cellular processes and metabolic functions, together with machine learning-assisted design and modelling, we may be able to combine these off-the-shelf to generate entirely novel genomes and organisms designed de novo for specific applications with vastly reduced turnaround times than any past and current genome writing projects^33,70^. Moreover, the simpler host can also be applied as a chassis to unravel the complexities of microbial ecosystems. With the growing capacity of genome synthesis and genome transplantation, it might be possible to incorporate a synthetic metagenome into a streamlined host cell. The resulting meta-synthetic cell will gain the traits from the ecosystem and have much improved industrial potential, with the metagenome containing all their keystone genes^71^. The meta-synthetic cell can also serve as a platform to study the communications and interactions of the eco-systems. Above all, synthetic minimal genomes could provide a path to inch closer to an answer to the genetic aspects of age-old questions: What constitutes life? To what extent can we reduce genomes while retaining industrial relevance? What is the trade-off between minimisation and application?
